# Molecular Cloning, Expression and Molecular Modeling of Chemosensory Protein from *Spodoptera litura* and Its Binding Properties with Rhodojaponin III

**DOI:** 10.1371/journal.pone.0047611

**Published:** 2012-10-25

**Authors:** Yanbo Zhang, Xiaolin Dong, Jinxiang Liu, Meiying Hu, Guohua Zhong, Peng Geng, Xin Yi

**Affiliations:** Laboratory of Insect Toxicology, Key Laboratory of Pesticide and Chemical Biology, Ministry of Education, South China Agricultural University, Guangzhou, People’s Republic of China; Russian Academy of Sciences, Institute for Biological Instrumentation, Russian Federation

## Abstract

Insects stimulate specific behaviors by the correct recognition of the chemicals in the external environment. Rhodojaponin III is a botanical grayanoid diterpenid oviposition deterrent isolated from *Rhododendron molle*. In this study we aimed to determine whether the CSPs involved in the recognition of Rhodojaponin III. A full-length cDNA encoding chemosensory protein was isolated from the antennae of *Spodoptera litura* Fabricius (CSPSlit, GenBank Accession No. DQ007458). The full-length cDNA of NlFoxA is 1789 bp and has an open reading frame (ORF) of 473 bp, encoding a protein of 126 amino acids, Northern blot analysis revealed that CSPSlit mRNA was mainly expressed in the antennae, legs, wings and female abdomens. A three-dimensional model of CSPSlit was constructed using homology modeling method, and its reliability was evaluated. The active site of CSPSlit was calculated using CDOCKER program indicated that the Tyr24, Ile45, Leu49, Thr64, Leu68, Trp79 and Leu82 were responsible ligand-binding active site on identifying Rhodojaponin III in the CSPSlit. The recombinant CSPSlit protein was expressed in *Escherichia coli* and purified using single-step Ni-NTA affinity chromatography. Fluorescence emission spectra revealed that the CSPSlit protein had significant affinity to rhodojaponin III. These results mean that CSPSlit is critical for insects identify the Rhodojaponin III.

## Introduction

Insects can recognize a variety of plant compounds that stimulate specific behaviors, such as feeding and egg laying (oviposition) by chemoreceptive organs [1]–[4]. It is well known that some insects lay eggs on their host plants, and the oviposition behavior is induced by the recognition of the plant components with sensilla on these chemoreceptive organs [5]–[6], But the detailed mechanism of this identification is not clear. Chemosensory proteins (CSPs) are a class of small (10–15 kDa) soluble proteins containing 4 conserved cysteines which abundantly exist in the chemoreceptive organs and transmit chemical signals to nervous system [7]–[8]. The CSP was first in *Drosophila melanogaster* and confirmed that CSPs are capable of binding a range of aliphatic compounds, esters and other long chain compounds that are typical components of pheromonal blends [7], [9].

The first member of the CSP family was discovered more than a decade ago in *Drosophila melanogaster* and was called olfactory specific protein D (OS-D) due to its preferential expression in the antennae [9]. Later studies identified other members of this family in sensory appendages such as antennae, labial palps and legs in a variety of insects [10]–[11]. Several members of this class of protein have been described in insects of different orders, including Lepidoptera [11]–[19], Orthoptera [10], [20]–[22], Hymenoptera [7], [23]–[26], Diptera [27], Blattoidea [28]–[29], Phasmatodea [30]–[32], Hemiptera [33], etc. The function of CSPs as carrier proteins was strengthened by studies on the higher order structure of a CSP from *Bombyx mori*, which revealed a globular configuration of six alpha helices surrounding a hydrophobic binding pocket [34]. Recent studies confirmed that CSPs are capable of binding a range of aliphatic compounds, esters and other long chain compounds that are typical components of pheromonal blends [7], [14]–[15], [35].

The *Spodoptera litura*, is one major pest of agricultural crops in many Asia areas. It is a polyphagous pest and known about 150 host species [36]–[37]. The extensively use of synthesis pesticides has caused it to develop resistance against various chemicals. The residual pesticides have not only polluted the environment, but are also a threat to human life. And it is serious during the seedling stage, especially in upland rice and other crucifer and it is also regarded as a very good target for the applications of rhodojaponin III [38]. Rhodojaponin III, a grayanoid diterpene compound isolated from the ﬂower of *Rhododendron molle*, has been reported to have high levels of oviposition deterrent, antifeedant, contact and/or stomach toxicity against more than 40 species of agricultural pests in laboratory bioassays and field trials [39]–[40]. However, the mechanism of rhodojaponin III as an oviposition deterrent is yet poorly understood.

The computer-aided structure-based study of molecular recognition is an important component of structure-based potential ligands screening [41]–[42]. The original DOCK algorithm addressed rigid body docking using a geometric matching algorithm to superimpose the ligand onto a negative image of the binding pocket [43]–[44]. A representative docking method is used to study these factors, namely, CDOCKER, a molecular dynamics (MD) simulated-annealing-based algorithm, which places a unique constraint on the development process [42].

The present study was designed to characterize and identify CSPSlit expression of the in Lepidoptera, *S. litura*, and the role of a grid representation of CSPSlit -rhodojaponin III interactions. We also intended to provide evidences to confirm the fundamental biological phenomena of CSPSlit and agricultural problems related to the *S. litura*.

## Materials and Methods

### 2.1 Insects and Preparation of Tissues

The *S. litura* were reared on an artificial diet [45], at 25±1°C in a 14:10 light: dark photoperiod and 60–70% relative humidity. Adults were harvested within 3 days after emergence. Different tissues (antennae, de-antennated heads, forelegs, mesopedes, metapedes, thoraces, wings and abdomens) were dissected from newly emerged adults and immediately frozen in liquid nitrogen and stored at −80°C until used.

**Figure 1:**
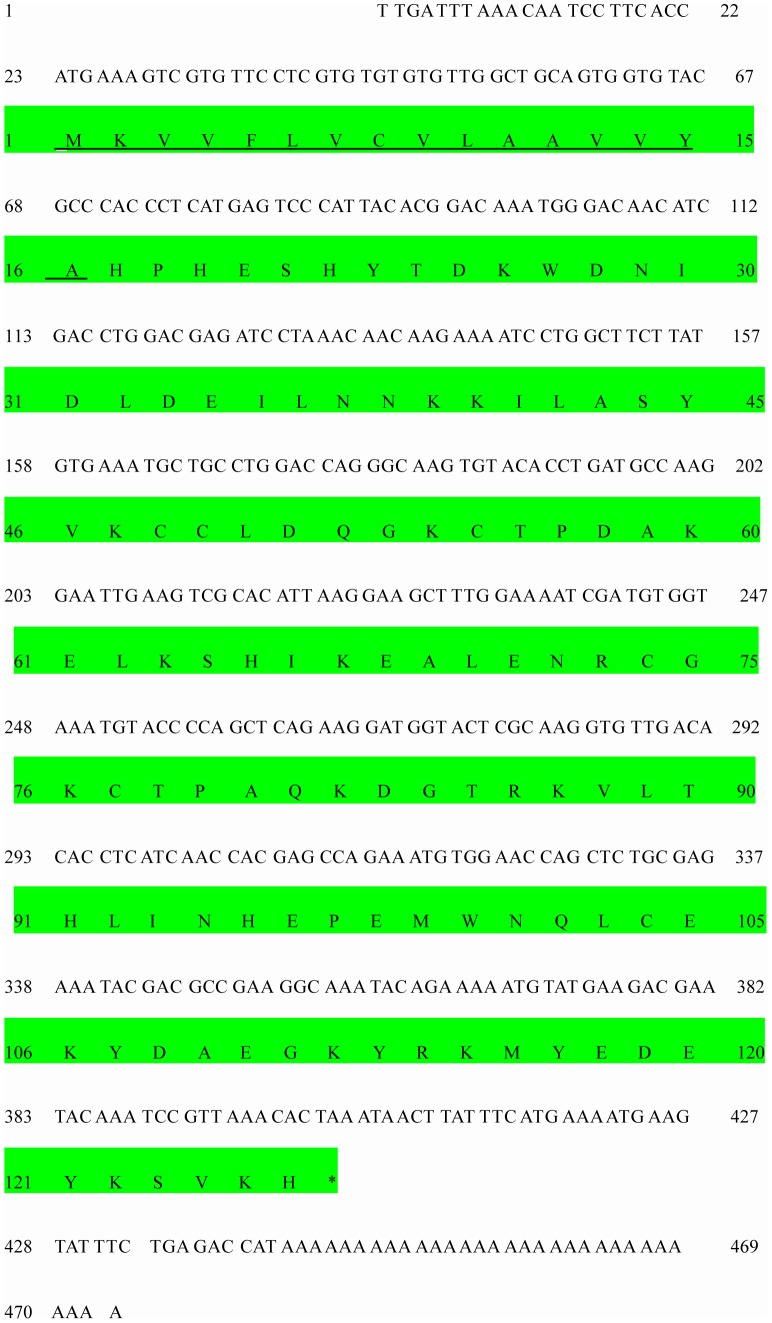
Nucleotide and deduced amino sequences of CSPSlit. The predicted signal peptide is underlined. The asterisk marks the translation-termination codon.

**Figure 2:**
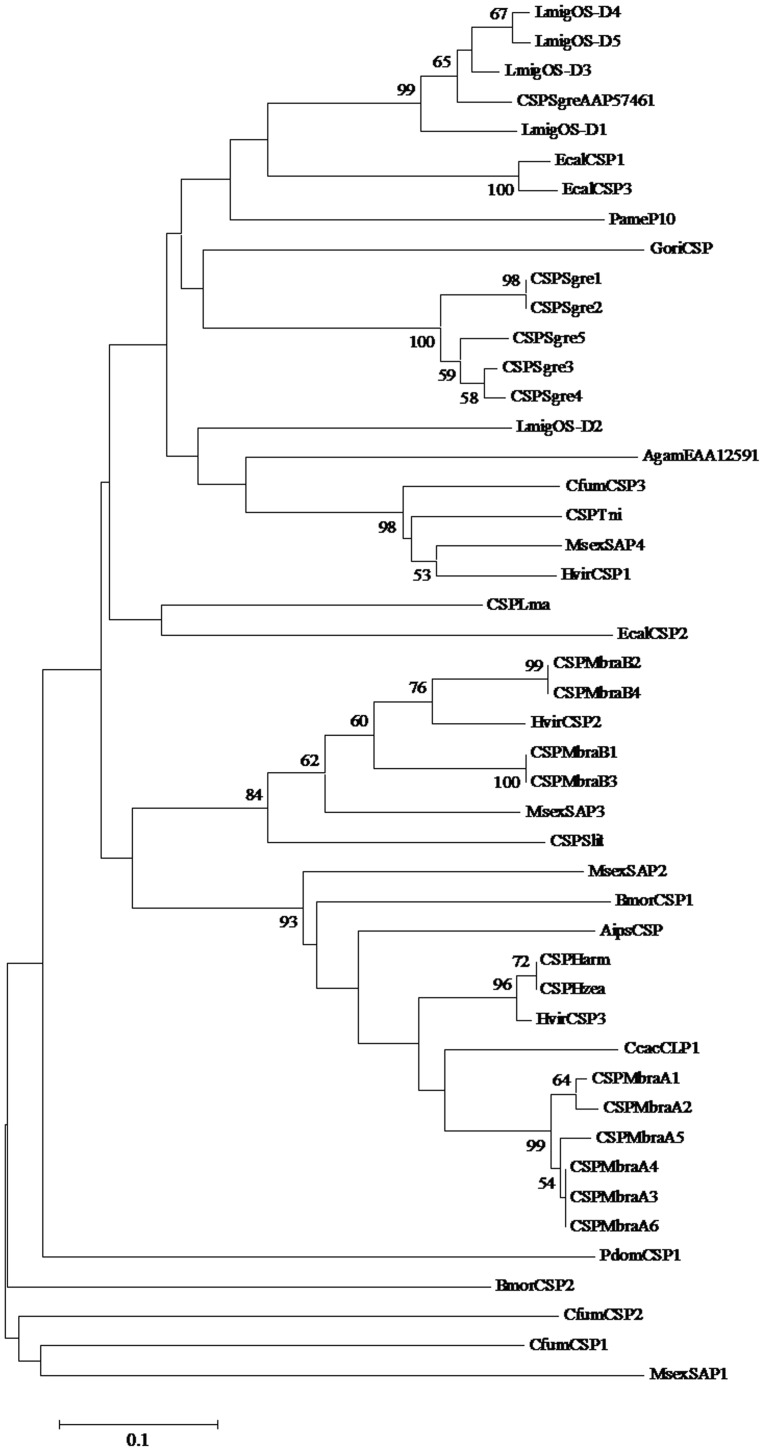
Phylogenetic analysis of CSP amino acid sequences. Bootstrap support values based on 1000 replicates are indicated.

### 2.2 Cloning and Sequence Analysis of CSPSlit

Cloning and sequence analysis of NlFoxA Total RNA was isolated from four 2 nd day brachypterous female adults of N. lugens using the Trizol kit (Invitrogen, USA). Its integrity was detected using Agilent 2100 Bioanalyzer (USA). First-strand cDNA was synthesized with a first strand synthesis kit using reverse transcriptase X L (AMV) and an oligo dT 18 primer (TaKaRa, Japan). Two pairs of degenerate primers were designed based on the conserved amino acid sequences of chemosensory proteins from different Lepidoptera insects. The first-strand cDNA (1 µl) was used as a template for PCR using a general protocol. The reaction mixture contained 0.1 mM dNTP, 0.5 mM of each degenerate primer and 1.0 U of HiFi-Taq DNA polymerase (TransGen Biotech, Guangzhou, China) in a total volume of 25 µl. The first PCR was carried out with the following conditions: initial preheating for 5 min at 94°C, 35 cycles at 94°C for 30 s, 48°C for 30 s and 72°C for 1 min, and with a final extension at 72°C for 10 min using the primer pair ACC GAC MRS TAY GAC AGY GAG AC and TCY TTG AGT TCC TTC TCR TAC TT. The second PCR was performed using another degenerate pair, CAA CCG YCG CCT SWT GGT GCY TAT and TAC TTG GCC KTC AGC TSK TTC CA, with the before mentioned program. The amplified fragment was recovered in a 1% agarose gel and purified using the Gel Extraction Kit (Omega, USA). Purified DNA was ligated into the pMD18-T vector (TaKaRa, Japan), and recombinant clones were digested with EcoRI and PstI to screen the presence of inserted DNA. Positive clones were sequenced by Invitrogen Company (Shanghai, China). To obtain the full-length NlFoxA cDNA, we used a RACE Kit (CLONTECH, Japan). Specific primers for the 5′- and 3′- Rapid Amplification of cDNA Ends (RACE) were designed based on homologous PCR fragments. The specific primers CGA CTT CAA TTC CTT GGC ATC AGG TG and GTT TAG GAT CTC GTC CAG GTC GAT G were used for 5′ –RACE, while GTA CCC CAG CTC AGA AGG ATG GTA C and GCC AGA AAT GTG GAA CCA GCT CTG C were used for 3′ –RACE. Using the 5′- and 3′-RACE cDNAs as templates, PCR was performed using the 5NlFoxA1 primer and Universal Primer Mix (UPM, Clontech) by denaturing at 95°C for 30 s, followed by 35 cycles of 95°C for 30 s, 55°C for 30 s and 72°C for 2 min, and a final extension at 72°C for 10 min. Nested PCR was carried out with the first-round PCR product as a template and the Nested Universal Primer A (NUP, Clontech) and NlFoxA2 primer. The reaction conditions consisted of the followings: 6 min of initial preheating at 94°C, 30 cycles of 94°C for 30 s, 68°C for 30 s and 72°C for 40 s, and a final elongation at 72°C for 7 min. The RACE products were purified and sequenced as described above. Sequence homologous alignment and similarity searches were carried out by Blast biological software http://www.ncbi.nlm.nih.gov/blast. The signal peptide was analyzed by SignalP procedure.

**Figure 3:**
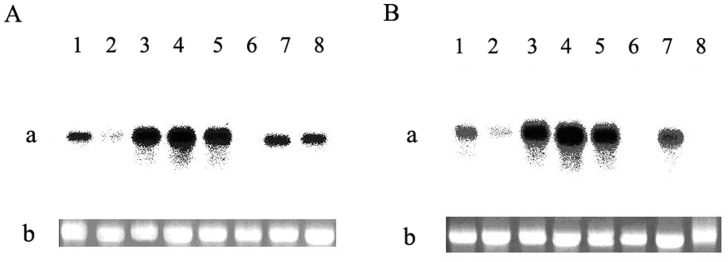
Northern blot analysis of RNA coding for CSPSlit in different tissues. (a): The recovery and integrity of each RNA were assessed from the 18S rRNA pattern; (b): 1, antennas; 2, de-antennated heads; 3, foreleg; 4, mesopedes; 5, metapedes; 6, thoraces; 7, wings; 8, abdomens. (A): female; (B): male.

**Figure 4:**
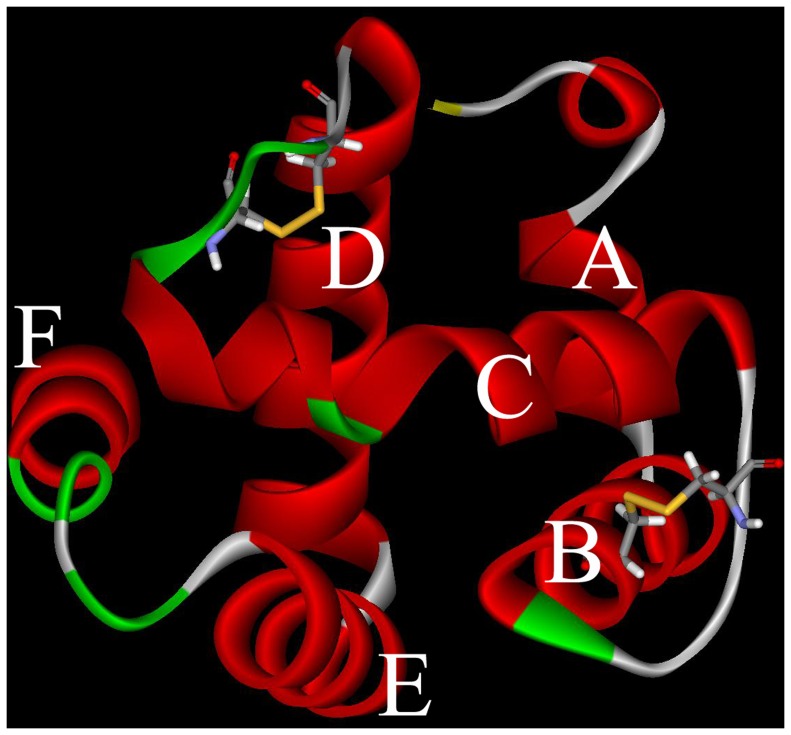
3D structure of the CSPSlit. Six α-helixes were marked A–F. Two disulfide bridges are represented by yellow stick.

**Figure 5:**
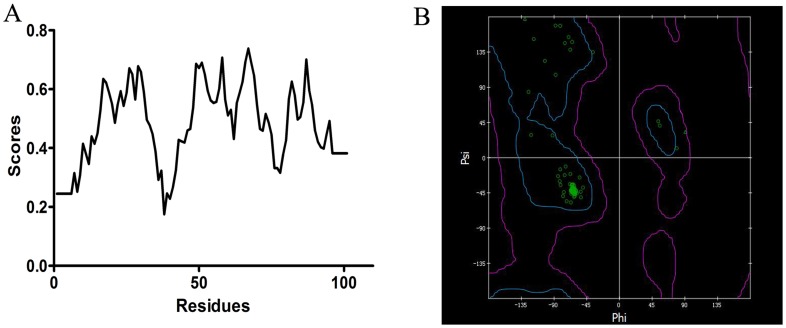
The evaluation of the CSPSlit 3D structure by verify score (A) and Ramachardran plot (B). Square: proline residues; triangle: glycine residues; circle: all other residues. blue and purple: favorable regions; all else: unfavorable regions.

**Figure 6:**
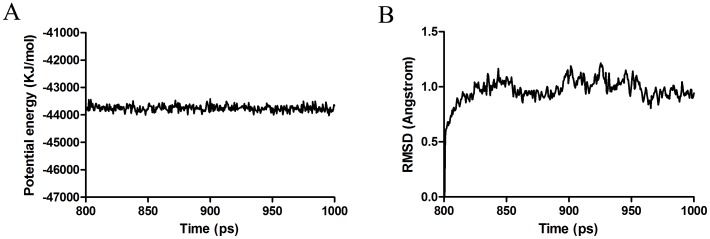
Potential energy (A) and root-mean-square deviation (A) with respect to simulation time for 1000 ps free MD simulation on the CSPSlit model.

### 2.3 Northern-blot Analysis

Total RNA was isolated as described above from the antennae, de-antennated heads, forelegs, mesopedes, metapedes, thoraces, wings and abdomens. Northern blot was carried out according to the method described by Sambrook [46]. Total RNA (20 µg/µl) was separated on 1.5% (W/V) denaturing formaldehyde agarose gels. The RNA was blotted onto NC membranes. The 405 bp fragment of CSPSlit was labeled with α-[^32^P]dCTP and used as a probe for hybridization at 68°C for 16 h. Final wash conditions for the RNA blots were 15 min at 68°C in 1×SSC, 0.2% (W/V) SDS, 15 min at 68°C in 0.5×SSC and 0.1% SDS. Washed membrane was dried at 80°C and exposed to X-ray film.

**Figure 7:**
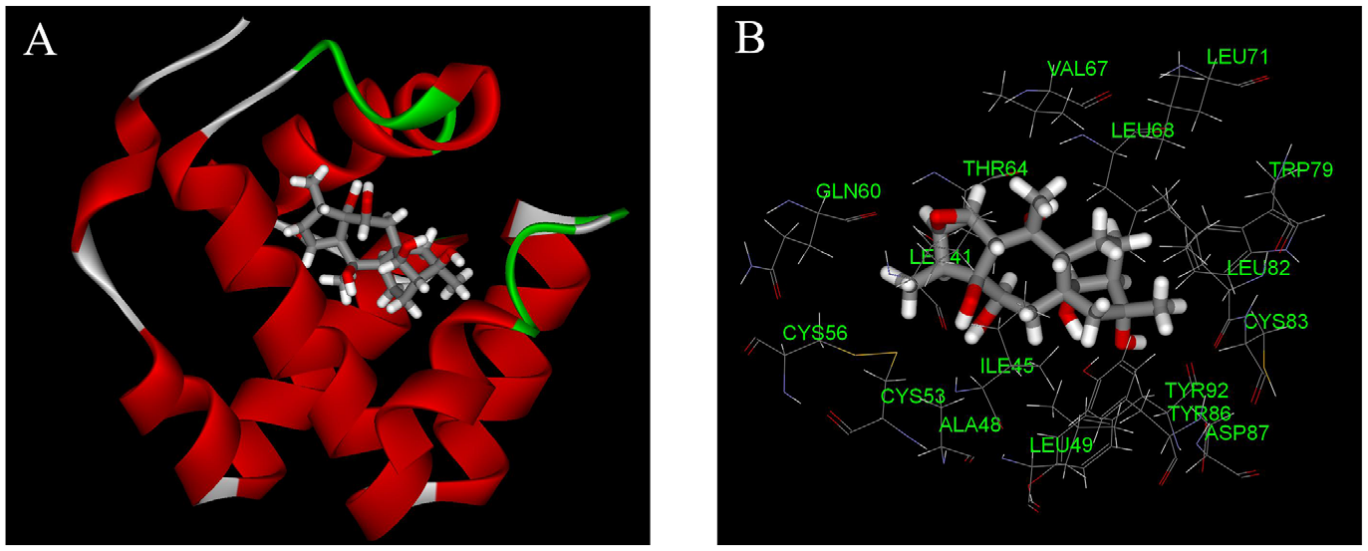
The complex (A) and detailed binding mode (B) of CSPSlit with rhodojaponin III. The residues within 6 Å from ligand are shown.

**Table 1:** VdW Energy (E_vdW_) and Electrostatic Energy (E_ele_) between rhodojaponin III and CSPSlit.

Residue	E_vdW_ (kcal/mol)	E_ele_ (kcal/mol)
Leu41	−0.830793	0.017039
Ile45	−3.700930	−0.346153
Ala48	−1.080190	0.739731
Leu49	−2.234000	−0.098858
Cys53	−0.358571	0.154808
Cys56	−0.318546	−0.240461
Gln60	−1.109890	0.331317
Thr64	−2.165880	−0.800064
Val67	−2.477020	0.549604
Leu68	−2.773280	−0.523853
Leu71	−0.885637	0.179657
Trp79	0.174320	0.187195
Leu82	−2.684090	0.219521
Cys83	−1.524360	0.297607
Tyr86	−1.640490	0.447274
Asp87	−0.697000	−0.607238
Tyr92	−0.572799	0.349116

**Figure 8:**
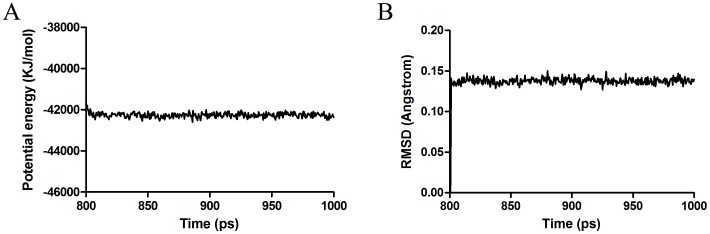
Potential energy (A) and root-mean-square deviation (B) with respect to simulation time for 1000 ps molecular dynamics simulation on the CSPSlit- rhodojaponin III complex model.

### 2.4 Expression and Refolding of CSPSlit

PCR was performed using the specific primers, 5′- CTG CCA TGG CAC ACC CAC ATG AG TCC-3′ and 5′- TTG AGT CTC GAG GTG TTT AAC GGA TTT G -3′ to obtain the open reading frame (ORF) of the CSPSlit. In order to facilitate sub-cloning of the ORF into the expression vector, the restriction sites of *Nco*I and *Xho*I were separately introduced into the forward and reverse primers. PCR products, purified by agarose gel electrophoresis, were digested with *Nco I* and *Xho I* enzymes and ligated into the *Nco I*/*Xho I*-digested pET-28a(+) (Novagen) to construct the expression vector pET-CSPSlit. The resultant plasmid was transformed into the competent *E. coli* BL21 (DE3). A single positive bacterial colony, which was confirmed by restriction enzyme digestion and sequencing, was inoculated into LB medium containing ampicillin (100 µg/ml) and grown overnight. The seed culture was diluted with 1∶100 of LB medium and grown at 37°C to the optical density of A_600_ = 0.4, and the cells were induced by addition of 0.5 mmol/L isopropyl-D-thiogalactoside (IPTG). After 6 h incubation at 28°C, cultures were harvested by centrifugation and lysed by the lysis solution (10 mM imidazole, 300 mM NaCl and 50 mM NaH_2_PO_4_). After sonication, the supernatants were recovered by centrifugation and subjected to the Ni2+-NTA column to purify the recombinant protein.

**Figure 9:**
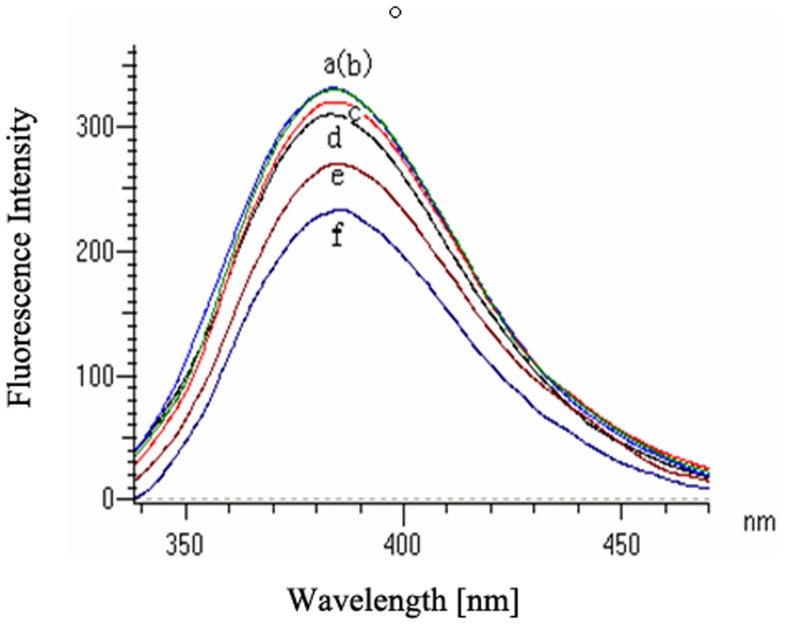
Fluorescence spectra obtained titrating a 0.5 µM solution of CSPSlit with increasing amounts of 50 µM rhodojaponin III in methanol to final concentrations of 0, 50, 100, 150, 300 and 600 µM (a–f). The intensity of this peak was used to measure the amount of rhodojaponin III bound to the protein.

The recombinant protein was refold with the approach of Tsumoto et al with some modified [47]. Brieﬂy, 10 ml recombinant protein was solubilized in denaturant buffer (6 M guanidine hydrochloride, 200 mM NaCl, 100 mM Tris-HCl, and 1 mM EDTA at pH 8.3). Denaturant was slowly removed by a series of overnight equilibrations with buffers of successive decreasing guanidine hydrochloride concentration. The guanidine hydrochloride concentration was reduced as follows: 6 M, 3 M, 2 M, 1 M, 0.5 M, 0 M. The 2 M guanidine hydrochloride equilibration buffer was supplemented with 400 mM L-arginine and 375 mM oxidized glutathione (GSSG) as folding additives. After the final overnight dialysis with buffer containing no denaturant, the sample was removed from the dialysis buffer and freeze-drying at −90°C, then stored at −80°C. The concentration of refolded CSPSlit was established with the Bradford method that a calculation based on the absorbance at 280 nm [48].

### 2.5 Molecular Modeling

The first step for modeling was to search for a number of related sequences to find the template structure which had a high sequence similarity with the target CSPSlit sequence on RCSB Protein Data Bank (PDB)(www.rcsb.org). The homology modeling of CSPSlit was performed using build homology model [49] protocol of Discovery Studio 2.0 (DS 2.0) (Accelrys Inc.). The best model was confirmed using the evaluation of PDF total energy, verify score and Ramachardran plot. After performing 1000 steps of steepest descend (SD) and 2000 steps of conjugate gradient (CG) minimization, a molecular dynamic (MD) simulation was carried out to examine the quality of the model structure, by checking its stability via performing 1000 ps simulations. An explicit solvent model TIP3P water was used [50], and the homology solvent model was constructed with a 20 Å water truncated octahedron from the center of CSPSlit mass.

### 2.6 Molecular Docking

The binding-site module is a suite of programs in DS 2.0 for identifying and characterizing protein binding sites and functional residues from protein. In this study, this program was used to search the protein binding site by locating cavities in CSPSlit structure, which can be used to guide the protein-ligand docking experiment. Molecular docking can fit molecules together in a favorable configuration to form a complex system. The structural information from the theoretically modeled complex may help us to clarify the binding mechanism between CSPSlit and rhodojaponin III. In order to gain insights into binding mode of CSPslit with rhodojaponin III, the advanced docking program CDOCKER [42] was used to perform the automated molecular docking. CDOCKER uses a CHARMm-based molecular dynamics scheme to dock ligands into a receptor binding site. Random ligand conformations were generated using high-temperature molecular dynamics. The conformations were translated into the binding site. Candidate poses were created using random rigid-body rotations followed by simulated annealing. A final energy minimization was used to refine the ligand poses. Finally, the docked complex of CSPslit with rhodojaponin III was selected according to the criteria of interacting energy combined with geometrical matching quality whereas the interaction energy between rhodojaponin III and key residues of CSPSlit was calculated. MD simulation was carried out to examine the binding quality of the complex structure, by checking its stability and performing 1000 ps simulations, as mentioned above.

### 2.7 Fluorescence Assays

In order to investigate the interaction of rhodojaponin III and CSPSlit, fluorescence quenching method was used to evaluate their binding character according to Lartigue [35]. The fluorescence spectra were recorded on a F-4500 FL Fluorescence Spectrophotometer (HITACHI) at 23°C. The interaction of rhodojaponin III and CSPSlit was monitored by following the quenching of the intrinsic protein fluorescence (excitation at 295 nm and emission 300–550 nm, slit width of 5 nm were used for both excitation and emission). Spectra were recorded with 0.5 µM protein in 10 mM Tris buffer, pH 7.4 and under the same conditions in the presence of different concentrations of rhodojaponin III (0, 50, 100, 300, and 600 µM).

## Results

### 3.1 cDNA Cloning and Sequence Analysis of CSPSlit

Two RACE fragments were amplified with four pairs of specific primers designed according to the nucleotide sequence of the fragment. By using rapid amplification of cDNA ends PCR (RACE-PCR), a full-length CSPSlit of 473 bp was obtained by overlapping the RACE fragments (GenBank Accession No: DQ007458). Sequence analysis showed that the full-length (ORF) of CSPSlit was 378 bp, encoding 126 amino acid residues, with a predicted MW of 14.67 kD. A 16-residue signal peptide in the CSPSlit was identified by SignalP, with a calculated molecular mass of a mature protein (110 amino acids) of 12.69 kD with an estimated pI of 6.66 by ExPASy [51] (Fig. 1). The phylogenetic tree was constructed based on the amino acid sequences CSP from *S.litura* and other insects (Fig. 2). The dendrogram showed that the CSPSlit had closer ancestry from the same order insects.

### 3.2 Tissues-specificity Expession Analysis of CSPSlit

To determine whether *CSPSlit* is present in various tissues in the *S. litura*, we used northern blot to characterize the pattern of tissues-specificity expression of *CSPSlit* gene from different tissues (male & female antennae, de-antennated heads, forelegs, mesopedes, metapedes, thoraces, wings and abdomens). Total RNA of each sample was isolated and separated, an approximately of 500 bps α-[32P]dCTP labeled CSPSlit antisense RNA probe gave strong hybridization signals to the antennae, legs and wings, lower trace was detected from de-antennated heads and thoraces, and it was expessed in female abdomen but absent in male (Fig. 3).

### 3.3 3D Modelling of CSPSlit Protein

The sequence of CSPSlit was compared to all known proteins in PDB and the results showed that chemosensory protein A6 from *Mamestra brassicae* (CSPMbraA6) (PDB code 1N8V) had the sequence identity (52%) with CSPSlit, so CSPMbraA6 was chose as template to model the 3D structure of the CSPSlit. Following the homology modeling, the best model (Fig. 4) was chosen from 10 candidates, and its quality was further checked by Ramachardran plot and verify score (Fig. 5). Figure 6 shows the time series of potential energy and root-mean-square deviation (RMSD) of backbone for 700 ps MD simulation of CSPSlit structure. The potential energy of the model was stabilized at 200 ps production after 800 ps equilibration and the RMSD of backbone compared to the starting coordinate remained at 1.0 Å up and down fluctuations. These 2 properties converged at production, indicating that the model is stable and can be used for subsequent docking calculation.

### 3.4 Molecular Interaction Analysis between Rhodojaponin III and CSPSlit

Considering the sequence conservation of CSPSlit with CSPMbraA6, the binding site was confirmed for its hydrophobicity. These binding poses were evaluated by score ligand pose program and ranked by Consensus score program. Finally, the optimal 3D binding conformations of complexes were selected and shown in Figure 7. The interaction energies between key residues and the ligand are listed in the Table 1. All of these residues are located in the cavity formed by six helices. Several residues including Ile45, Leu49, Thr64, Leu68 and Leu82 identified by current docking simulations have been shown to play an important role in the binding of CSPSlit and rhodojaponin III. Figure 8 shows the time series of potential energy and RMSD of backbone for 1000 ps MD simulation of CSPSlit structure. The potential energy of the complex was stabilized at 200 ps production after 800 ps equilibration, and the RMSD of backbone compared to the starting coordinate remained at 0.14 Å up and down fluctuations. These two properties converged at production, indicating that the complex is stable.

### 3.5 Binding Assay of Rhodojaponin III and CSPSlit

Purified CSPSlit protein solution contained 5.0 mg/ml was used to analyse the binding property of CSPSlit with rhodojaponin III. When excited at 295 nm, the fluorescence emission spectra showed maximally relative fluorescence intensity at 383.6 nm for CSPSlit. Following, with the increasing concentration of rhodojaponin III, CSPSlit peak underwent a blue shift, but no peak intensity increaseing was observed. When the final concentration of rhodojaponin III was 600 µM and 300 µM, the fluorescence intensity decreased to 29.43% and 18.47%, respectively (Fig. 9). These results showed that CSPSlit could be intensely bound with rhodojaponin III.

## Discussion

The olfactory system of insects is essential for Lepidoptera as well as in other insect orders to initiate behavioral responses, such as searching for food sources, mating, oviposition and feeding [52]–[53]. Chemosensory proteins (CSPs), known as another class of soluble protein, share no sequence homology with either PBPs or general OBPs of many insects [11]–[12], [18]. The CSPs are smaller proteins which contain four cysteines instead of six with conserved interval spacing involved in two disulfide bonds [35], [54]. In the present study, a cDNA sequence encoding the CSP of *S. litura* was cloned. The CSPSlit has 4 typical conservative cysteines in the sequence (Fig.1). It is consistent with previous report. The *CSPSlit* was expressed in antennae, legs, wings and female abdomens (Fig.3), these results is similar with the research in other insect [9], [21]. In *M. brassicae*, the CSPs has a abundant expression in proboscis [15], [30], but in this study, there is no hybridization signal was detected in the de-antennated head (Fig. 3). These results indicated that the CSPs could not only recognize the odours but tastes. The result of Northern blot in this study revealed that the CSPSlit was found not only in antennas but also in legs, deantennated heads, thoraces, wings and abdomens. Especially in legs, its specifically high expression suggested that CSPSlit was perhaps functionally associated with contact chemoreception. CSPSlit with preferentially expression in female abdomen but absent in male suggested that CSPSlit might involve in female-specific chemical senses during mating or oviposition.

Homology modeling is based on the assumption that the proteins with similar sequences might have analogous 3D structures. Thus, selection of a suitable template is the first step. The structural characterization of CSPMbraA6 by NMR for the first time; The crystal structure of CSPMbraA6 in complex with one of these compounds, 12-bromo-dodecanol, reveals extensive conformational changes on binding, resulting in the formation of a large cavity filled by three ligand molecules [8]. However, in the absence of an experimentally determined crystal structure, it is generally recognized that homology modeling of proteins is currently the most accurate method for 3D structure prediction [55]–[58]. To study the binding mechanism of rhodojaponin III-CSPSlit complex in depth, the best way is to acquire the 3D crystal structure of it. In PDB, CSPMbraA6 was chosen as the template for constructing the 3D model of CSPSlit for it has 52% amino acid sequence identity with CSPSlit. To assess the reasonable of the 3D model of CSPSlit, Ramachandran plot, verify score and molecular dynamic were used [59]–[60] and showed positive results (Fig. 5, 6). The results of quality assessment suggest that the model of CSPSlit structure is of reasonable quality compared to the crystal structure of the CSPMbraA6 complex [8].

The 3D model of CSPSlit showed that CSPSlit had typical structure of CSPs,six α-helices has been described in 3D structure of CSPSlit, and four of six cysteine residues are conserved in sequence alignments and formed 2 disulphide bridges (C1–C3 and C4–C5), which enforce the organization of the helices [7]–[8], [34]–[35]. All helices are amphiphilic, with the hydrophobic sides formed mostly by leucines and isoleucines. In 3D model of CSPSlit, helices A and B as well as helices D and E form two V shaped structures as a ‘binding pocket’. Helix C is perpendicular to these two planes and positioned in between the four ends of the two V-shaped structures to close one end of this pocket. The final helix (F) is located packed against the external face of the D-E helices and does not take part in the core assembly. This cavity is delimited by the hydrophobic sides of these helices, and therefore well-suited to constitute a binding site for hydrophobic ligands [7]–[8], [15], [20], [34].

The interaction energies between the rhodojaponin III and the important residues of CSPSlit are listed in Table 1. Although the interaction energies we obtained in the current docking study do not reproduce the real binding free energies, the relative values should be meaningful [59], [61]. Our docking results indicated that Ile 45 could have strong hydrophobic interactions with rhodojaponin III. The residue in this position has been shown to affect substrate binding or specificity in CSPSlit. Analysis of interactions between rhodojaponin III and CSPSlit reveals that vdW energy has a larger contribution to ligand binding than the electrostatic energy. This is in line with the fact that the binding pocket of CSPSlit is mainly composed of hydrophobic residues [7]–[8], [15], [34]. The Tyr 24 side chain might be rotated towards the protein surface like a door to open or close the entrance of the cavity. Although the Tyr 24 is not involved in the key residues we predicted in the binding of rhodojaponin III and CSPSlit, it might be play the role as mentioned above. The interaction of Tyr 24 and rhodojaponin III might occur before the ligand coming into the cavity. When this happened, the Tyr 24 is rotated and exposed the cavity to rhodojaponin III. Another interaction occurs after rhodojaponin III was imbedded in the cavity and the door will closed. Rhodojaponin III is fully imbedded and fit well in the cavity. It positions with the hydrophilic group turned to the outside are also in agreement with the hydrophobic character of the channel [8], [35], [34]. The hydrogen atoms of the rhodojaponin III are closer to the Trp 79 indole rings than to Trp 6. This orientation accounts well for the fluorescence of Trp 79 being mostly quenched. Our docking results were in good agreement with the experimental data. The more details in the binding modes of CSPSlit and its substrates need further investigations through combination of molecular, genetics and chemical methods.
